# Effect of the Juggling-Based Motor Learning Physical Activity on Well-Being in Elderly: A Pre–Post Study with a Special Training Protocol

**DOI:** 10.3390/healthcare10122442

**Published:** 2022-12-03

**Authors:** Jakub Malik, Janusz Maciaszek

**Affiliations:** Department of Physical Activity and Health Promotion Science, Poznan University of Physical Education, Królowej Jadwigi 27/39, 61-871 Poznań, Poland

**Keywords:** well-being, depression, cognitive development, fall prevention, exercise, elderly, motor learning, juggling, physical activity, exercise protocol

## Abstract

Background: The importance of physical activity for the elderly is undeniable. Specific forms of exercise that are able to engage practitioners, both cognitively and physically, may provide more positive consequences for health and quality of life. Juggling is one of these activities that has both of these characteristics. Methods: Twenty elderly people (70.55 ± 4.91) were included in a juggling-based motor learning protocol for twelve training units during one month of exercising. An evaluation of the proposed exercises (five-point Likert scale) and a subjective assessment of well-being (WHO-5) were conducted during the protocol. Results: All participants learned to perform a three-ball flash cascade. Exercises were rated as very attractive (4.85 ± 0.31) by the practitioners, and a statistically significant improvement in well-being in participants was shown (*p* < 0.01; d = 0.76). Additionally, in the participating group, the number of people at risk of depression decreased significantly after the intervention with juggling classes (*p* < 0.01; g = 0.5). Conclusions: The proposed protocol could be an interesting physical activity for the elderly. It can be assumed that this activity, especially when performed in a group form, can improve the well-being of participants in a short period of time.

## 1. Introduction

Population studies have shown that cognitive and motor functions decline similarly during the aging process. Between 45 and 65 years of age, the decline is linear, but after age 65, the effect is rapid [1]. However, as described by Murman [2], the changes that constitute and influence aging are complex. The progressive dysfunction of the central nervous system, peripheral nervous system and neuromuscular system with age appears to be the main effect of motor deficits in the elderly [3]. Cognitive dysfunction is also caused by age-related changes in the neuronal structure. The risk of these changes increases with age, and age-related diseases may accelerate the rate of neuronal dysfunction and loss [2]. These deficits make it difficult for the elderly to perform activities of daily living and can be one of the main factors that increase the risk of falls, which are very dangerous for the elderly [3]. The main causes of motor and cognitive deficits include the deterioration of coordination skills, the increased variability of movements and difficulties with balance, processing speed, working memory, executive functions and others [2,3]. However, despite the progressive changes with age, the aging process should be viewed as heterogeneous because it depends on many factors, such as comorbidities, intrinsic abilities (cognitive and physical), the environment or functional abilities. Therefore, in the opinion of Nguyen et al. [4], the process of aging and the weakening of cognitive as well as physical abilities will progress but in an individualized way in each adult.

One of the proven methods for developing cognitive functions in people is physical activity [5,6,7,8,9,10,11,12,13]. The promotion of physical activity, especially among the elderly, has become a potent strategy to improve their well-being. Regular physical activity is linked to mental health, especially acting as a preventive measure for depression or neurodegenerative diseases [5]. It not only can improve attention, memory and executive functioning but also may be a means of preventing cognitive impairment [6]. An active lifestyle and regular physical activity or exercise might also improve motor performance and the motor learning process [7]. In addition, other findings showed that combining physical exercise and cognitive training into one activity leads to greater improvement in cognitive functions in the elderly [8]. This type of exercise increases the hippocampus [9], which is involved in motor learning [10], and in addition, exercises involving the cardiovascular system increase the activity of the sensorimotor network [11] or blood flow in the motor cortex, which is responsible for motor control [12]. Moreover, cognitively demanding activities, such as motor learning, do not need to affect cardiorespiratory fitness to have a beneficial effect on cognition and sensorimotor performance [13]. These findings are promising for our aging society. For this reason, motor learning deserves special attention.

Motor learning is “…a set of processes associated with practice or experience leading to relatively permanent changes in the capability for responding” [14]. As we have presented in our systematic review [15], the evidence shows that in the elderly, motor learning retains the ability to change the brain structure. Older adults achieve a worse final performance, but the neuroplastic changes in the brain are similar to those of younger people with a better final performance of juggling. There is still no conclusive knowledge about whether these changes persist long-term or affect the day-to-day functioning of the elderly.

Nevertheless, this evidence seems promising enough to make it worthwhile to introduce activities that focus strictly on motor learning in the elderly. One example of such a form of activity is classical juggling. This is a form of activity that requires concentration while tossing and catching balls in a specific sequence. The number of possible combinations of techniques is so large that even with a long training period, practitioners can learn new tasks during each meeting. Evidence suggests that juggling among the elderly can induce neuroplasticity and thus perhaps improve cognitive and motor functions, and importantly, the elderly are still able to master juggling [15]. These changes can occur after just one week of a juggling intervention [15,16].

However, in addition to the ability to function physically or cognitively, a sense of well-being is also important to health-related quality of life [17]. Worse mental well-being is associated with an increased risk of physical illnesses [18,19], but positive mental well-being works as a protective factor [19,20]. Stewart and King [19] concluded that physical activity correlated with well-being and turned out to be the most important element linking mental well-being and human health. Existing evidence suggests that older people’s participation in exercise increases their sense of self-efficacy, which in turn affects their well-being [21]. In addition, physical activity can have a positive impact on mood and life satisfaction [22,23] by reducing, among other things, symptoms of depression in the elderly [24]. So, it is worth encouraging older adults to take up different, especially new, forms of activity. Additionally, in order to be able to reach a wider community of older adults, it is also necessary to offer them a wide range of these activities.

Due to the limited requirements of juggling, it seems to be a good form of activity for people who do not have the opportunity to leave the house or are forced to stay there for a certain period of time, for example, due to quarantine. However, there is a lack of research evaluating the impact of juggling on aspects of daily life, such as its effect on participants’ cognitive function and mood. In addition, the lack of protocols for practicing this activity causes people, especially older adults, to not try juggling, so they miss out on the benefits that juggling can bring.

The purpose of this study was to implement the authors’ juggling-based motor learning protocol (J-BMLP) in the elderly, evaluate its quality and assess the impact of the J-BMLP on the subjective well-being of participants. Our hypothesis is that after physical activity in the form of juggling, there will be a positive change in the perception of the well-being of the participants.

## 2. Materials and Methods

### 2.1. Participants

Twenty volunteers (mean age = 70.55 ± 4.91) who responded to a local advertisement were included in the single-arm pre–post study design with the J-BMLP intervention. Advertisements were published on the local radio, television and newspapers and also as ads on social networks covering the reach of the city of Poznań, located in Poland. The exclusion criteria for participation in the J-BMLP were as follows: individuals with mixed or left-handedness, any neurological impairments, any balance issues, prior experience in juggling or contraindications to physical activity and individuals under 65 years of age. Participants were asked not to take up new forms of activity during the project, but their previous activities were not restricted during the study, and their physical activity levels were not recorded. Right-handedness was only advisable for more efficient juggling classes. The characteristics of all participants are included in Table 1.

### 2.2. Assessment of the Attractiveness and Well-Being

The J-BMLP was assessed by participants individually after completing the whole protocol. For this purpose, a simple proprietary evaluation scale for proposed activities was used. This scale is based on five-point Likert ratings, where participants’ opinions about the difficulty, cognitive effort, motor effort and attractiveness of exercises were assessed. A rating of 5 on the scale indicated high attractiveness, effort or difficulty, while a rating of 1 meant low attractiveness, effort or difficulty. Participants filled out the J-BMLP evaluation questionnaire anonymously. Additionally, before and after the J-BMLP, participants were asked to respond to the Polish version of the Well-Being Index (WHO-5), which assesses subjective well-being, referring to the last two weeks. This index can identify depressive symptoms in elderly subjects. An optimal cutoff value of <13 points proves to be the most accurate value in identifying depression. WHO-5 showed good internal and external validity [26,27]. For the Polish adaptation of the WHO-5 scale, Cronbach’s alpha was 0.87, while convergent validity was reported as good: (a) correlation with PHQ-9 r = 0.75; (b) negative correlation with PAID r = 0.52 [27]. Each time, before beginning the questionnaires, participants were instructed on how to complete the WHO-5 scale by the same researcher. The researcher did not interfere in the process of filling out the scale. Participants were not limited by time.

### 2.3. J-BMLP Description

The main goal of the J-BMLP was to learn a juggling trick named the three-ball cascade. The elderly can learn cascade juggling with three balls, which can improve their cognitive and motor functions (or prevent the impairment of these functions) when the activity is regularly continued [11,28]. The proposed exercise protocol is designed for the elderly as an additional and interesting physical activity that can be performed individually in the privacy of one’s house or in organized classes. For the purposes of the study, the protocol was conducted as a group class with a qualified juggling instructor.

The J-BMLP included twelve training units, with each class lasting 45 min (a warm-up and a cool-down were included). Exercising and learning the steps of the three-ball cascade were included in the main part of the training unit. Each exercise of this protocol could have been modified by increasing its difficulty, e.g., clapping hands during a throw or a toss or directing attention to objects in front of the participant. For the purpose of this study, each task was performed for 3 to 5 min. In all of these exercises, it was important to involve both the dominant and non-dominant sides of the body. Participants in each exercise started tossing or throwing balls with the dominant hand and then with the non-dominant hand. The entire protocol consists of three main groups of exercises called column exercises, various exercises and parabola exercises.

**Figure 1:**
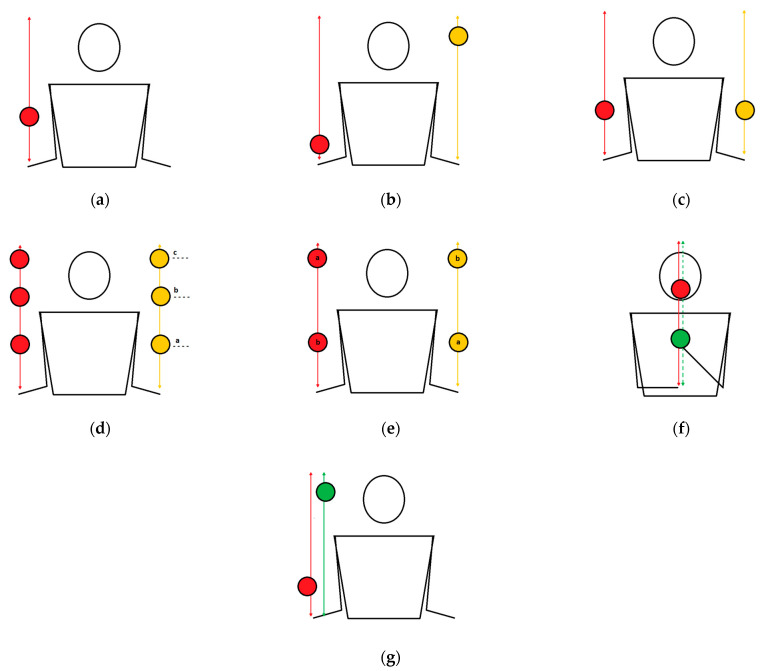
Column juggling exercises: (**a**) the one-handed column; (**b**) two-handed alternate columns; (**c**) two-handed simultaneous columns; (**d**) two-handed simultaneous columns at three levels; (**e**) two-handed simultaneous columns at different levels; (**f**) fake columns; (**g**) two-balls-in-one-hand columns.

Column exercises are a type of juggling exercise and require the straight vertical trajectory of a ball when thrown (Figure 1). During these exercises, the focus should be placed on the ball, not on the individual hand position. The toss and catch should be performed by the same hand. Balls in these exercises (with some exceptions) should be tossed at eye level. With a higher toss, it is difficult to maintain the correct trajectory of the balls, but exercisers have more time to react.

- The one-handed column is the simplest task of this type. Using one hand, practitioners should toss and catch one ball.
- The two-handed alternate-column task requires tossing balls in alternating order. The task should be performed smoothly; i.e., tossing one ball should occur when the other is at the peak of its trajectory.
- The two-handed simultaneous columns is a task in which exercisers should toss two balls using two hands simultaneously at the same level. Attention should be directed toward both balls at the same time.
- The two-handed simultaneous column task at three levels requires throwing balls using two hands at the same level in a specific order: (1) the first (low), (2) the second (medium, eye line); (3) the third (high) level.
- The two-handed simultaneous columns at different levels is a task in which practitioners should toss two balls in the following order: (1) the dominant hand—the first level; the non-dominant hand—the third level; (2) the dominant hand—the third level; the non-dominant hand—the first level.
- Fake columns are a group of similar tasks that require tossing and isolation exercises at the same time. Isolation is not throwing but guiding the ball using a hand along the trajectory of a column, just like a tossed ball. The ball, which is guided by a hand, can be next to, above or below the tossed ball. It is important to react with an isolated ball to the movements of the tossed ball.
- The two-balls-in-one-hand column task requires tossing two balls alternately in the same hand. Each toss should be performed when the previous ball is at the peak of its column trajectory.

**Figure 2:**
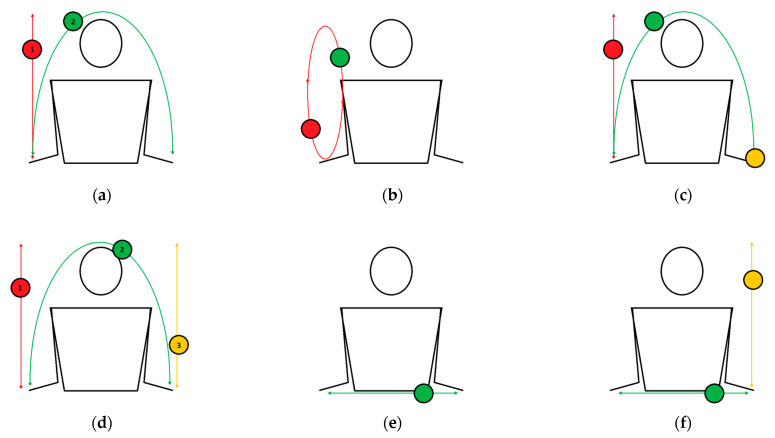
Various juggling exercises: (**a**) the one-handed column and the parabola; (**b**) the two-balls-in-one-hand fountain; (**c**) the one-handed column and the parabola with three balls at pace; (**d**) the three-ball “W”; (**e**) the zero throw; (**f**) the two-ball box.

Various exercises are a group of tasks that require more than one style of throw (Figure 2). It should be based on previously learned tasks. The main purpose of these various tasks is to prepare for more demanding tasks and also maintain the motor learning process to make previously learned exercises more difficult. Therefore, these exercises should be implemented between the column and parabola tasks. Throws should be performed mainly at eye level.

- The one-handed column and the parabola task is a combination of the one-handed column and the one-handed parabola. Two balls are in one hand. During one repetition, exercisers should follow the correct order: (1) toss the first ball in a column trajectory; (2) throw the second ball in a parabolic trajectory; (3) catch the first ball; (4) catch the second ball.
- The two-balls-in-one-hand fountain is a task where balls move in an ellipse. Two balls are held in one hand. The rhythm of tossing is important: when one ball is at the peak of its trajectory, then the practitioner should toss the second ball. The purpose of this exercise is to master the correct rhythm of tossing the balls. It is not necessary to catch both balls in the same hand. If someone performs this exercise well, we encourage them to try to perform as many such tosses as possible in a series.
- The one-handed column and the parabola with three balls at pace is a task that differs from “the one-handed column and the parabola” with a third ball. Two balls should be held in one hand, with the third in the other. The task should start from the hand with two balls. The order of performing this task is as follows: (1) toss the first ball in a column trajectory; (2) throw the second ball in a parabolic trajectory; (3) catch the first ball; (4) catch the second ball with the hand in which the third ball is held.
- The three-ball “W” task is a more difficult version of the previous task and requires smooth movement so that none of the balls come into contact with each other.
- The zero throw is a simple task that involves the dynamic throwing of a ball in a horizontal line from hand to hand.
- The two-ball box task requires simultaneously moving two balls in the following order: (1) a column toss of the first ball and a zero throw of the second ball at the same time; (2) a zero throw of the second ball and a catch of the first ball at the same time. This task can be performed alternately on both sides at pace.

**Figure 3:**
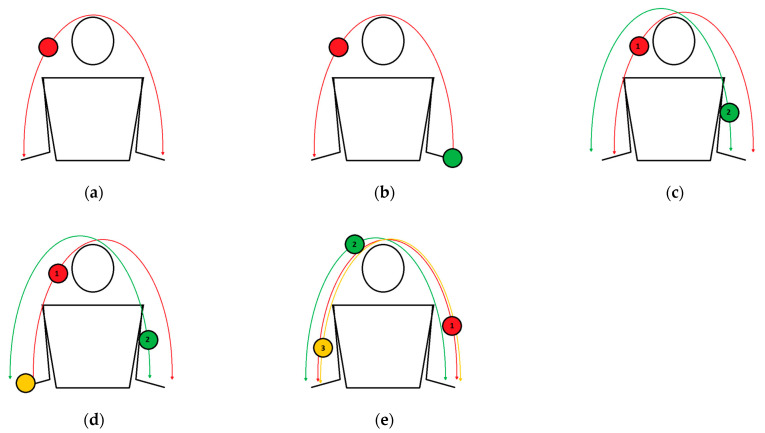
Various juggling exercises: (**a**) the one-handed parabola; (**b**) the one-handed parabola, with the second ball in a catching hand; (**c**) two-handed parabolas with a different focus of attention; (**d**) two-handed parabolas with three balls; (**e**) the zero throw.

Parabola exercises are a group of tasks in juggling that require a throw from one hand to the other (Figure 3). The throw should be performed at the eye line along a parabolic trajectory. With two or more balls, each successive throw should be performed while the previous ball is at the peak of its trajectory. It is important to avoid synchronously throwing the balls. Attention should be focused on the ball that is in the air, not on the position of the hands or other balls.

- The one-handed parabola is the simplest task of the parabola exercises and involves throwing the ball from hand to hand. Exercisers should avoid the direct transfer of the ball.
- The one-handed parabola with the second ball in the catching hand task requires throwing a ball from one hand to the other. The exercisers should complete one repetition of this exercise with two balls in one hand. An important progression in this exercise is to release the second ball before the first ball lands in the hand, as follows: (1) throw the first ball; (2) release the second ball on the ground; (3) catch the first ball.
- The two-handed parabolas with a different focus of attention is a group of tasks with two balls (each in one hand), where it is required to throw balls in the following order: (1) throw the first ball; (2) throw the second ball; (3) catch/ignore the first ball; (4) catch/ignore the second ball. The practitioner should focus their attention only on the first ball and ignore the second ball or on the second ball and then ignore the first ball. Ignoring is understood as letting the ball fall to the ground. It is necessary to have the perfect parabolic trajectory of both balls. The ball on which the participant focuses their attention must be caught. The goal of this task is to throw and catch both balls alternately at pace.
- The two-handed parabolas with three balls/throwing two balls is a more difficult version of the previous task. This task requires alternating the throwing and catching of both balls at pace with an additional ball constantly held in a hand. The practitioners should always start the task from the hand with two balls.
- The two-handed parabolas with three balls with a different focus of attention is a group of tasks that require exercisers to throw three balls along a perfect parabolic trajectory. In this task, practitioners should start from the hand with two balls. The order of movement in this task should be as follows: (1) throw the first ball; (2) throw the second ball; (3) throw the third ball; (4) catch/ignore the first ball; (5) catch the second ball; (6) catch/ignore the third ball. Ignoring is understood as mentioned previously—letting the ball fall to the ground. In one version of this task, the first two balls should be caught, and the third can fall; in the second version, the first ball can fall, and the second and the third should be caught. A difficult variance of this task is a “flash” cascade with three balls, which means that each ball should be thrown and caught once at pace. In this task, exercisers should try to throw the balls alternately along a parabolic trajectory and catch them all. One repetition of this task should proceed in the following order: (1) throw the first ball; (2) throw the second ball; (3) throw the third ball; (4) catch the first ball; (5) catch the second ball; (6) catch the third ball. This task is also the aim of this protocol. The next step in this task is to juggle continuously for a long sequence. An easier version of this task consists of throwing three balls in a parabolic trajectory but focusing on only one of them (the first, the second or the third), and this ball should be caught, while the other two may fall to the ground.

The whole J-BMLP is included in the Supplementary Materials (see additional written material (Supplemental Digital Content 1), which describes the whole protocol of the juggling intervention for people with no previous experience in this activity and includes the step-by-step motor learning process; videos (Supplemental Digital Content 2–66) are provided that demonstrate each exercise described in the additional written materials of Supplemental Digital Content 1. For the purpose of this research, all training units were conducted by the same person. The instructor was a person characterized by experience in teaching physical education in schools and in conducting sports gymnastic classes for children. The person had several years of juggling experience.

### 2.4. Statistical Methods

For the purpose of this study, we used the W Shapiro–Wilk test for assessing the normality of variables. In the next step, a paired-sample *t*-test for measuring differences in dependent variables (before and after protocol) in the Well-Being Index results was used. We also used the dichotomization of the values of WHO-5 to divide participants into those at risk for depression and those not at risk for depression. For categoric variables, we used the McNemar test to assess the impact of the intervention on the risk of a depressive state in participants. All statistical analyses were performed using STATISTICA (version 13.3.0, TIBCO Software Inc, Palo Alto, CA, USA; 2017). Cohen’s d formula was used to calculate the effect size for the paired-sample *t*-test (where 0.2—small effect; 0.5—moderate effect; 0.8—large effect), and Cohen’s g formula was used to calculate the effect size for the McNemar test (where 0.05—small effect; 0.15—moderate effect; 0.25—large effect) [29].

### 2.5. Sample Size Calculation

The sample size was calculated in G*Power software (Version 3.1.9.6, Düsseldorf, Germany) [30]. The “means: difference between two dependent means (matched pairs)” test from the *t*-test family for a priori estimation was used. According to a minimal power of 0.8, an alpha error of probability of 0.05, and an effect size of 0.732 [31] for depression assessment after moderate activities, a sample size of 14 participants is adequate. Additionally, due to the ongoing risk of COVID-19 infection among participants, 40% more people were recruited, which amounted to 20 participants.

## 3. Results

All of the included participants were able to perform the three-ball flash cascade. With this protocol, the elderly, on average, achieved the three-ball flash cascade skill after seven training units.

**Figure 4:**
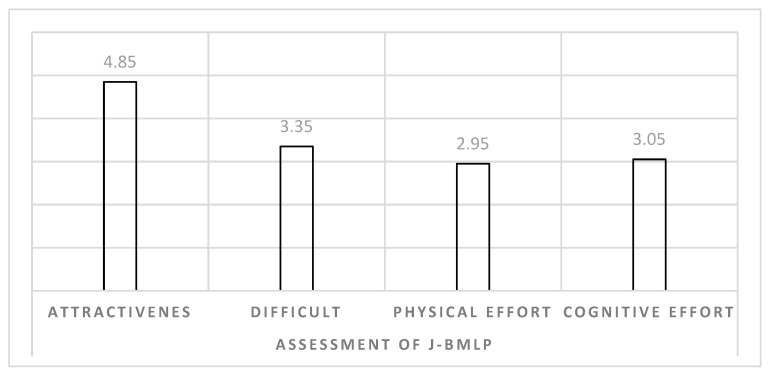
Participants’ assessment of J-BMLP.

The attractiveness of the project, the difficulty of the tasks and the physical and cognitive efforts were rated by the elderly participants of the juggling class (Figure 4). On the rating scale, the protocol was found to be very attractive (4.85 ± 0.37), moderately difficult (3.35 ± 0.75) and moderately demanding in terms of both physical (2.95 ± 0.76) and cognitive effort (3.05 ± 0.69).

**Figure 5:**
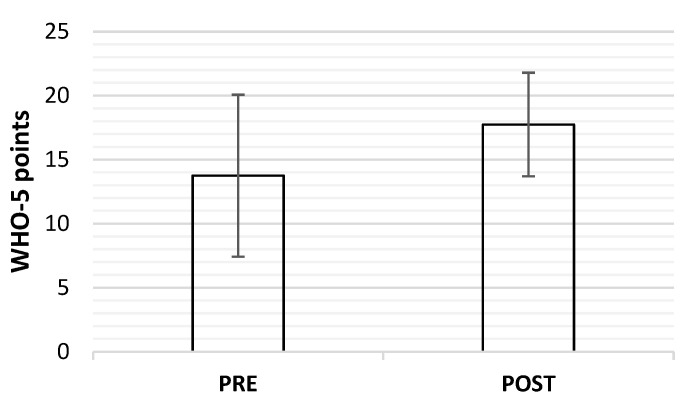
Difference in subjective assessment of participants’ well-being before (PRE) and after (POST) the intervention presented as averages with standard deviation. Statistically significant difference *p* < 0.01; d = 0.76.

The average of the WHO-5 values for all participants before the start of the protocol was close to the sensitivity value of 13.00 points (WHO-5_before_ = 13.75 ± 6.32). After the protocol, the average had improved (WHO-5_after_ = 17.75 ± 4.04), and this difference is statistically significant and shows a moderate effect size (*p* < 0.01; d = 0.76). The data are shown in Figure 5.

Authors of the Well-Being Index suggest that a score below 13 indicates malaise and is an indication for an examination for depression according to the ICD-10 classification. Before the J-BMLP, 50% of participants classified themselves as depressed under these criteria. After the protocol, just 5% of participants scored below 13 points. Additionally, a WHO-5 result 10% higher than the previous value for a single subject, according to the scale key, indicates a significant improvement in well-being [32]. Such a result was obtained by 60% of the participants. For 20%, no change was perceived. For 5%, the subjective assessment of well-being increased slightly. For the remaining 15%, it decreased significantly, but none of these individuals scored below the sensitivity threshold. After dichotomizing these variables (A: <13 points—risk of depressive state; B: ≥13 points—no risk of depressive state), it turns out that the effect of the intervention on the probability of a depressive state in older adults is statistically significant with a large effect size and effectively reduces this risk (*p* < 0.01; g = 0.5). The dichotomous data of the WHO-5 scale are cross-tabulated in Table 2.

## 4. Discussion

The purpose of this study was to test the J-BMLP as a physical activity proposal for the elderly that may have the potential for developing cognitive function and coordination abilities among participants. As the results show, along with its moderate difficulty and moderate cognitive and physical effort is the high attractiveness of this protocol. It seems that juggling proves to be an interesting form of physical activity even for people over 65 years old. The high value of this protocol is that it does not require specialized equipment and can be performed in a standing or sitting position due to the involvement of only the upper body. Additionally, the proposed exercises can be safely performed without the presence of an instructor and can be performed by the elderly with positive performance results, and it is not necessary to have a large space to perform the recommended exercises, so there is no obstacle to juggling alone at home.

The main problem in promoting physical activity programs is understanding the motives and barriers involved. Following McLeroy’s socio-ecological model, three main criteria for barriers and motivators regarding physical activity in seniors can be mentioned: intrapersonal, interpersonal and environmental [33,34]. According to numerous studies, the main motivator for older adults to engage in physical activity is to improve their physical condition [34,35,36]. In contrast, the second most important intrapersonal motivators are pleasure [34,35] and solving psychological problems (such as relief from stress or depression) [34,36,37]. Participation in the J-BMLP seems to give a lot of pleasure to the participants. Among all evaluations, the program received a rating of “attractive” or “very attractive”. The assessment of well-being and, consequently, the classification of participants as at risk or not at risk for depression also changed positively after the J-BMLP intervention, which may provide an added incentive for potential beneficiaries to participate in this program. Assessing changes in overall physical fitness was not the goal of this research; however, our current research confirms reports that the elderly can successfully start learning juggling [28,38]. Mastering a new, complex skill in a relatively short period of time may have had an impact on increasing their confidence in their own abilities and in their physical fitness. Studies show that elderly people are physically active achieve higher levels of self-esteem and self-efficacy [39,40,41]. When it comes to interpersonal motivations for older adults to engage in physical activity, sociability is of the greatest importance. This is understood as communication with others, peer support or exercising together [35,39,42,43]. This shows the advantage of conducting the J-BMLP as a group class with an instructor. Although the J-BMLP is safe to do on one’s own without an instructor present, it is likely that the effect of such exercises would not be as high as in a group class. This is supported by studies showing the advantage of group exercise over individual exercise on mental health [37,44]. The main environmental motivator for physical activity for seniors is a group of elements related to the environment in which the elderly person is active. Such an environment should be attractive, with pleasant landscapes, well lit and free of uncultured social activities [34,43,45,46]. In our study, the J-BMLP intervention took place in a sports hall, but these exercises can be performed in any environment, even where space is limited (such as at home). A larger space and, above all, a natural environment, such as a park, would certainly increase the attractiveness of this type of activity. There is no obstacle to performing the proposed J-BMLP exercises outdoors.

As the evidence shows, an active lifestyle can have a positive impact on an individual’s self-esteem due to improved levels of independence, which are associated with utility, leisure activities, physical autonomy and perceived health in old age, among other things. Consequently, engaging in physical activity enhances self-esteem [41] and thus can contribute to an improved quality of life [39,47]. Our research shows a large effect size of the implemented protocol as an exercise intervention, which reduces the chances of being placed in a risk group in the WHO-5 depression screening test. The main reason for this phenomenon is the moderate effect size of the improvement in subjective well-being on the WHO-5 questionnaire after one month of participation in J-BMLP group activities. Current evidence [37,44] shows the superiority of group exercise over individual exercise in the fight against depression. In contrast, the loss of opportunities to interact directly with people in physical activity classes is associated with an increase in depressive symptoms. We believe that the main reason for the improvement in WHO-5 scores among the participants in our study was the group format of the classes.

Unfortunately, it is not always possible to take part in group activities. For example, the pandemic situation, which required restrictions and isolation, made it difficult to undertake physical activity at all. The frequency of pandemics is increasing, and this trend is likely to continue [48]. So, situations such as those that occurred during the COVID-19 pandemic can still be expected. As the evidence shows, such situations cause a decline in physical activity levels among people of all ages and an increase in sedentary behaviors [49]. In such cases, higher levels of physical activity and exercise also contribute to greater well-being [47,50], despite the inability to participate in group activities. One of the suggestions in such conditions may be the J-BMLP because of the limited requirements for space or equipment (after all, participants can juggle anything). The instructional videos included in the Supplemental Digital Content can be helpful when direct contact with the instructor is not possible. An additional strength of the J-BMLP may be that case study research has shown that juggling as a form of therapy also has a beneficial effect on treating refractory PTSD symptoms [51]. In addition, other studies have noted that regular juggling can also effectively reduce anxiety [52]. There is a suggestion that the temporal lobe of the brain is involved in generating panic attacks [53], so movement information and physical movement itself may be an important part of controlling this problem. Existing evidence suggests that juggling as a physical activity that engages the practitioner’s attention precisely affects the neuroplasticity of the brain, both white [15,54,55] and gray matter [15,16,28,56,57], including but not limited to the medial temporal lobe [15,16,28,56,57,58]. This shows that juggling as a physical activity can effectively improve well-being. This may be due to a form of activity that requires focus, such as some form of meditation, or due to its neuroplastic property.

Based on the existing premises and this research, the J-BMLP can be considered an interesting form of physical activity for the elderly. The proposed exercises included in the Supplementary Materials can also be incorporated into various training units as an attractive form of activity or be modified for the purposes of the training unit. If these exercises are to be performed on a special separate training unit, a warm-up is necessary to prepare the exercisers for the effort. The “milestones” of the proposed three-ball cascade juggling process are described in specific groups of juggling tasks and with increasing difficulty. It should be remembered that the motor learning process depends on different variables and can work differently in people of the same age [59,60].

This protocol, for those interested, can serve as an introduction to learning other juggling tricks in the future. The exercises included in the protocol can be undertaken by anyone who has no contraindications to physical activity. However, certain medical conditions may hinder the performance of these exercises due to the requirement of precise tosses and catches with the upper extremities.

## 5. Limitations

The main limitation of the research conducted is the lack of a control group to draw cause-and-effect conclusions from the measurements. In addition, the J-BMLP activity self-reported evaluation scale, which is based on the 5-point Likert scale, represents a measurement of participants’ opinions rather than explicit variables and thus should not be compared with other reliable measurements of the feeling of physical or cognitive fatigue. Despite the high strength of the effect, and thus the calculated sample size, it seems that studies conducted on a larger number of participants would allow inferences of greater external validity. In addition, the participants were a group of volunteers and thus an unrepresentative group of people relative to the whole elderly population. Thus, one can speculate that J-BMLP classes were attended by people interested in this form of activity, which may have influenced the final rating of the attractiveness of these classes.

## 6. Conclusions

Although our data suggest that the offered physical activity based on juggling and motor learning can improve subjective ratings of well-being, the study’s methodology does not make it clear what influences this change. Of the many variables, such as decreased COVID-19 restrictions, the effect of socialization during the activity or even life changes that may have occurred during the project, it is difficult to isolate the J-BMLP as the one of primary importance.

Nevertheless, the large effect size of the changes and the ease of carrying out the exercises of the J-BMLP under almost any conditions allowed us to infer that it is worth implementing this form of physical activity for the elderly with the expectation of positive results in both physical fitness and well-being.

Further research should be based on randomized controlled trials with a larger number of participants, with an attempt to isolate any side and confounding variables, so as to be able to demonstrate the actual impact of a specific physical activity on the well-being of the elderly.

## Figures and Tables

**Table 1:** Characteristics of participants.

Variable	PRE Mean ± SD	POST Mean ± SD
Age	70.55 ± 4.91	-
Body mass [kg]	67.55 ± 14.11	67.85 ± 13.58
Height [cm]	161.50 ± 8.79	-
BMI [kg/m^2^]	25.82 ± 3.77	25.97 ± 3.73
Handedness ^1^	91.88 ± 13.27	-

^1^ Values from 100 to 61—right-handedness; 60 to −60—mixed handedness; −61 to −100—left-handedness; the Edinburgh Handedness Questionnaire—short form [25].

**Table 2:** Cross-tabulated WHO-5 subjective well-being rating scale data before (PRE) and after (POST) the intervention.

	POST		
PRE	WHO-5 < 13 Points [n]	WHO-5 ≥ 13 Points [n]	Total
WHO-5 < 13 points [n]	1	9 *	10
WHO-5 ≥ 13 points [n]	0 *	10	10
Total	1	19	20

* Statistically significant improvement, *p* < 0.01; g = 0.5.

## Data Availability

Not applicable.

## Supplementary Materials

The following supporting information can be downloaded at: https://www.mdpi.com/article/10.3390/healthcare10122442/s1, Training protocol: Supplemental Digital Content 1; Videos: Supplemental Digital Content 2–66.
