# Supplementary material for: Effect of the Juggling-Based Motor Learning Physical Activity on Well-Being in Elderly: A Pre–Post Study with a Special Training Protocol
Source: Healthcare (Basel). 2022 Dec 3;10(12):2442. doi: 10.3390/healthcare10122442 (PMC9778107; doi:10.3390/healthcare10122442)
Supplement: Supplementary file 1 [file healthcare-10-02442-s001.zip › Supplemental Digital Content 1.pdf]

The Juggling intervention for people with no previous experience in this activity including step-by-step motor learning process. The main goal of this protocol is to achieve the ability to perform the most popular juggling trick – the three ball cascade. This protocol contains twelve specific unis.

45 minutes of one training unit consisted of three phases:

Warm-up	Juggling activity	Calm-down
8 minutes	32 minutes	5 minutes

Warm-up

It was a regular part of the training units. The purpose of the warm-up was to prepare practitioners for the exercise and, in addition, to familiarize with juggling instruments. The first training unit required performing standing exercises during the warm-up. The next units required performing standing exercises, marching in place in rhythm and movements of the upper limbs. Examples of exercises used in the warm-up are listed below:

- Synchronous and asynchronous shoulder flexion and extension with ball transfer in front
- Synchronous and asynchronous shoulder abduction and adduction with ball transfer over the head
- Releasing the ball into an open hand (one hand is placed higher than the other one)
- Arm circles
- Elbow circles
- Arms waving – up, down with ball transfer in front
- Arms waving - diagonally with ball transfer in front
- Synchronous combination of moves in following order: shoulder flexion, adduction, abduction, extension
- Moving arms to the side and froward with ball transfer in front
- Torso twist
- Ball transfer under a knee (the knee is high bended)
- Hip circles
- Ball transfer around the hips
- Stretch neck exercises
- Wrists circles
- Lifting the toes
- Etc.

Juggling activity

If a task requires you to hold two balls in one hand, it is best to hold one ball with your little and heart finger, while holding the other ball with your thumb and middle and index finger.

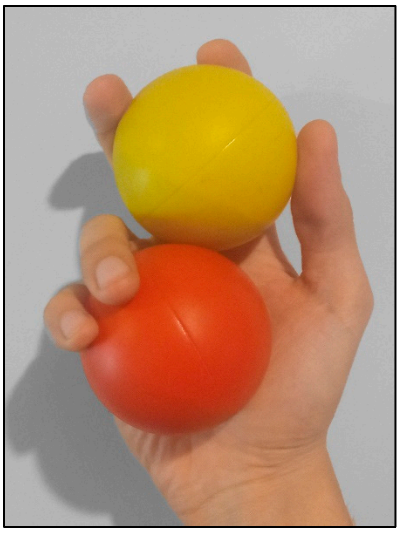

The main part of the training unit. It mostly consisted of two stages: perfecting previous elements and learning new elements. Each task should be performed with right and left hand equally. The best way is to start tossing with the dominant hand, and then with the nondominant hand. The following table shows the course of the main part:

Unit	Exercises	Explanation
**First unit**<br><br>**(only learning new elements)**	**Learning:**<br><br>***1.*** **The one-handed column**<br>*(see video, Supplemental Digital Content 2, which demonstrates exercise named: "the one-handed column")*<br><br>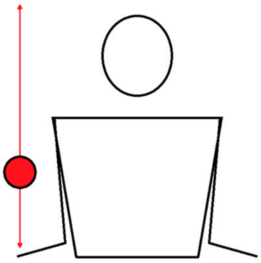<br><br>**2. The one-handed column with clapping hands**<br>*(see video, Supplemental Digital Content 3, which demonstrates exercise named: "the one-handed column with clapping hands")*	**1.**<br>The column with one hand. It's important to focus on the trajectory of the ball. It should be in a straight line. Practitioners should toss the ball at the eye level or higher. The higher the toss, the more difficult it is to maintain a proper trajectory. It is important to focus on the ball trajectory – not on the position of the hand.<br><br>**2.**<br>In this exercise practitioners should be able to toss the ball in a column in a straight line and simultaneously clap their hands before the ball drops in a hand.

	**3. The one-handed column with tapping on the thigh**<br>*(see video, Supplemental Digital Content 4, which demonstrates exercise named: "the one-handed column with tapping on the thigh")*	**3.**<br>It's another version of *no. 2.* but here practitioners should tap on the thigh when the ball is in the air.
	**4. The one-handed column, catching the ball in the second hand**<br>*(see video, Supplemental Digital Content 5, which demonstrates exercise "the one-handed column, catching the ball in the second hand")*	**4.**<br>The importance of this exercise is tossing the ball in the straight trajectory. The second hand (the one that did not toss the ball) should reach for the ball. If the ball is thrown toward the hand that is supposed to catch it, it is a mistake.
	**5. The one-handed parabola**<br>*(see video, Supplemental Digital Content 6, which demonstrates exercise named: "the one-handed parabola")*<br>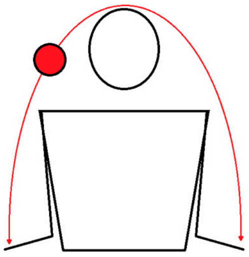	**5.**<br>The parabola with one ball is a simple exercise, where practitioner should throw the ball into the second hand. The eye level is a perfect high of the throw. It is important to focus on the trajectory of the ball, not on the hands' position. A big mistake is a direct transfer of the ball from hand to hand. The practitioners should avoid this by throwing the ball.
	**6. The one-handed parabola at pace**<br>*(see video, Supplemental Digital Content 7, which demonstrates exercise named: "the one-handed parabola at pace")*	**6.**<br>The difference in this exercise in relation to *no. 5.* is to throw the ball at pace. The time of holding the ball should decrease significantly. Throwing should be done from hand to hand repeatedly.
	**7. The one-handed column with a double circle movement**<br>*(see video, Supplemental Digital Content 8, which demonstrates exercise named: "the one-handed column with a double circle movement")*	**7.**<br>It's a very similar exercise to *no. 1.* Here in addition practitioners should perform a double circulate movement of the hand, which will immediately perform the throwing. In order to create a good habit, the circular motion should be performed twice before throwing. It's important to keep a straight trajectory of the throw. The circle movement should be performed like an outward movement (look at the figure).

	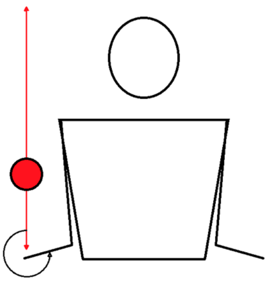	
	**8. The one-handed column, with a double circle movement, while catching the ball with the second hand**<br>*(see video, Supplemental Digital Content 9, which demonstrates exercise named: "the one handed column with a double circle movement, and catching the ball with the second hand")*	**8.**<br>Exercise similar to *no. 4.* In addition practitioners should perform a double circle movement.
	**9. The one-handed parabola, with a double circle movement**<br>*(see video, Supplemental Digital Content 10, which demonstrates exercise named: "the one-handed parabola with a double circle movement")*	**9.**<br>It's a very similar exercise to *no. 5.* In addition practitioners should perform a double circle movement.
	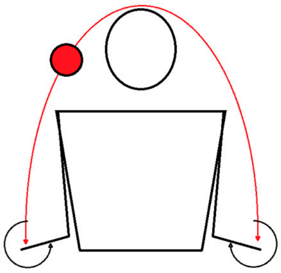	
	**10. The one-handed parabola, with a circle movement at pace**<br>*(see video, Supplemental Digital Content 11, which demonstrates exercise named: "the one-handed parabola, with a one circle movement at pace")*	**10.**<br>It's a standard parabola exercise with one hand. But now practitioners should perform just one circle movement with the hand, which will immediately perform the throwing. This movement is important for giving the ball the right force and trajectory during a parabola throw. With a larger number of balls, this movement helps to maintain a proper throwing rhythm.

	**11. The one-handed parabola, with a circle movement at pace, during a march**	**11.**<br>It’s the same exercise as the previous one. with addition of a march movement. Practitioners should perform this juggling exercise marching three steps back and forth.
**Second unit**	**Practicing:**<br><br>**1. The one-handed column**<br>**2. The one-handed column with clapping hands**<br>**3. The one-handed column with tapping on the thigh**<br>**9. The one-handed parabola, with a double circle movement**<br>**10. The one-handed parabola, with a circle movement at pace**<br><br>**Learning:**	**Practicing phase**<br>It is a phase of the main part of the training units, where practitioners perform previously learned exercises.
	**12. The one-handed parabola at pace with tapping on the chest**<br>*(see video, Supplemental Digital Content 12, which demonstrates exercise named: “the one-handed parabola in pace with tapping on the chest”)*	**12.**<br>A standard one-handed parabola exercise with an additional tap on the chest. Here it is important to follow a specific order of the tasks: (1) throw the ball with one hand in a parabola trajectory, (2) tap on the chest with the second hand, (3) catch the ball with the second hand (the one that made a tap). Practitioners can count on to make the task easier. Performing the tasks at the same time (e.g., throw and tap) is a mistake and should be corrected.
	**13. The one-handed parabola in pace with tapping on the chest and a circle movement**<br>*(see video, Supplemental Digital Content 13, which demonstrates exercise named: “the one-handed parabola in pace with tapping on the chest and circle movement”)*	**13.**<br>Similar to *no. 12.,* with additional circle movement of the hand which should immediately perform the throw. The order of the movement is as follows: (1) one circle movement by the hand with the ball, (2) throw the ball in a parabola trajectory, (3) tap on the chest with the second hand, (4) catch the ball with the second hand (the one that made a tap). Performing these tasks simultaneously is a mistake. Practitioners can count to make the task easier.

	**14. The two-handed simultaneous columns at three levels**<br>*(see video, Supplemental Digital Content 14, which demonstrates exercise named: "the two-handed simultaneous columns at three levels")*<br>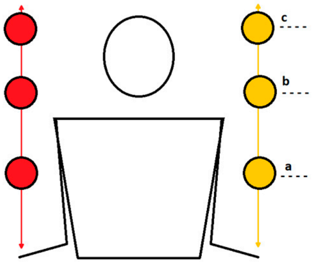	**14.**<br>Two balls, each in different hand. Exercise where practitioners should toss balls simultaneously with left and right hand. It's important to perform the throws in a straight trajectory. The order of tossing should be as follows: (a) the first (low), (b) the second (medium, eye line), (c) the third (high) level. In this exercise attention should be focused on two ball simultaneously.
	**15. The two-handed simultaneous columns at different levels**<br>*(see videos, Supplemental Digital Content 15 and Supplemental Digital Content 16, which demonstrate exercises named: "the two-handed simultaneous columns at the two different levels" and "the two-handed simultaneous columns at the three different levels")*<br>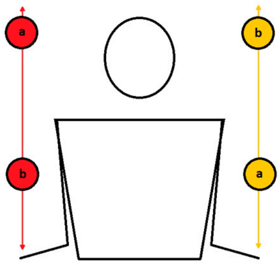	**15.**<br>Two balls, each in different hand. Each hand should perform simultaneous toss using different strength. A straight trajectory is still very important. Order of this exercise should be as follows: (a) dominant hand – the first level, nondominant hand – the third level; (b) dominant hand – the third level, nondominant hand – the first level. Tossing should be performed at pace – short time of holding the balls.
	**16. The two-handed alternate columns with arms' extensions**<br>*(see video, Supplemental Digital Content 17, which demonstrates exercise named: "the two-handed alternate columns with arms' extension")*	**16.**<br>Two balls, each in different hand. Colum is performed by both hands. This exercise is very similar to *no.1.* The difference is that practitioners should focus once on one ball, once on the other one by tossing them alternately. Straightness of trajectory is still very important. During first attempts, practitioner should throw out once one and once the other one, at individual pace. After every catch and before every throw – arms should be extended. To perfect this exercise practitioners should toss balls

	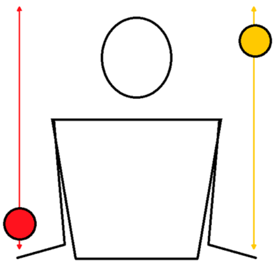	alternately at such pace that the throw of the second ball occurs when the first ball reaches its peak (both hands are empty for some time). In this more difficult version of this exercise, an extension of arms is not necessary.
	**17. The two-handed alternative columns at different levels**<br>*(see video, Supplemental Digital Content 18, which demonstrates exercise named: "the two handed alternate columns at different levels")*	**17.**<br>Two balls, each in different hand. This exercise is very similar to the previous one. Extension of arms should not occur here. Practitioners should toss a ball alternately in the following order: (a) dominant hand – the first level, nondominant hand – the first level; (b) dominant hand – the second level, nondominant hand – the second level; where the second level is described as eye line. Attention is focused only on the ball which is in the air. Straight trajectory is important.
	**18. The two-handed alternative columns with a tap on the thigh**<br>*(see video, Supplemental Digital Content 19, which demonstrates exercise named: "the two-handed alternate columns with a tap on the thigh")*	**18.**<br>Two balls, each in different hand. Level of tossing in this exercise is standard – the eye line. Practitioners should perform tossing the balls alternately with straight trajectory. After the throw, the same hand should tap on the thigh, and in next step catch the falling ball.
	**19. The two-handed alternative columns with a circle movement and a tap on the chest**<br>*(see video, Supplemental Digital Content 20, which demonstrates exercise named: "the two-handed alternate columns with a circle movement and the tap on the chest")*	**19.**<br>Two balls, each in different hand. In this exercise in comparison to *no.18.* practitioners should perform a tap on the chest with the same hand which just tossed the ball, and at last catch the ball.
	**20. The two-handed alternative columns with marking**<br>*(see video, Supplemental Digital Content 21, which demonstrates exercise named: "the two-handed alternate columns with marking")*	**20.**<br>Two balls, each in different hand. Classic alternative column with two hands. Nevertheless in this exercise one hand does not toss, but makes the move needed to toss a ball, but does not release it from the hand. Order of this task is as follows: (a) one hand throws the ball, (b) the second

	**21. The one-handed parabola, with the second ball in the catching hand**<br>*(see video, Supplemental Digital Content 22, which demonstrates exercise named: "the one-handed parabola, with the second ball in the catching hand")*<br>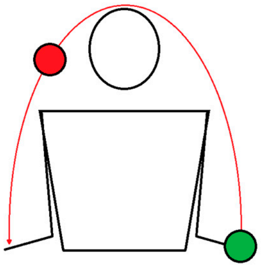	hand marks the throw, (c) the first hand catches the ball.<br><br>**21.**<br>Two balls, each in different hand. In this exercise practitioners hold two balls, but perform the throw in the parabola trajectory by just one of them. The second ball is the whole time in the second hand making it difficult to catch the first ball. After grabbing two balls in one hand, one of the balls must be discarded so that each hand is again holding only one ball. It is a mistake to transfer the ball directly from hand to hand.
**Third unit**	**Practicing:**<br><br>**14. The two-handed simultaneous columns at three levels**<br>**15. The two-handed simultaneous columns at different levels**<br>**16. The two-handed alternative columns with arms' extensions**<br>**20. The two-handed alternative columns with marking**<br>**21. The one-handed parabola, with the second ball in the catching hand**<br><br>**Learning:**<br><br>**22. The one-handed parabola, with the second ball in the catching hand, releasing the second ball**<br>*(see video, Supplemental Digital Content 23, which demonstrates exercise named: "the one-handed parabola, with the second ball in the catching hand, releasing the second ball")*	**Practicing phase**<br>It is a phase of main part of training units, where practitioners perform previously learned exercises.<br><br>**22.**<br>Two balls, each in different hand. Practitioners should be focused only on the first ball. Order of the movement is important: (1) throw the first ball, (2) release the second ball on the ground, (3) catch the first ball. The first ball should be thrown in a parabola trajectory to the other hand. It does not matter how practitioners release the second ball, it's important to catch the first ball in the empty hand.

	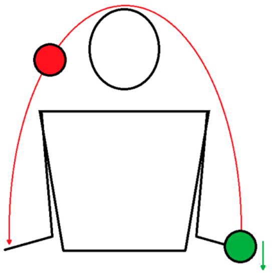<br>**23. The one-handed parabola, with the second ball in the catching hand, marking**<br>*(see video, Supplemental Digital Content 24, which demonstrates exercise named: "the one-handed parabola, with the second ball in the catching hand, marking")*	**23.**<br>Two balls, each in different hand. The exercise is very similar to the previous one. Here practitioners should follow the following sequence: (1) throw the first ball, (2) marking with the second ball in hand, (3) catch the first ball (both balls in one hand).
	**24. The two-handed parabolas with a different focus of attention – catching the first ball**<br>*(see video, Supplemental Digital Content 25, which demonstrates exercise named: "the two-handed parabolas with a different focus of attention – catching the first ball")*	**24.**<br>Two balls, each in different hand. In this exercise practitioners should focus on the first ball. The second ball should be thrown in a parabola trajectory, but it is not necessary to catch the second ball. The first ball should be thrown in a perfect parabola trajectory and caught in the second – empty hand. The order of this exercise: (1) throw the first ball, (2) throw the second ball, (3) catch the first ball, (4) the second ball falls on the ground.
	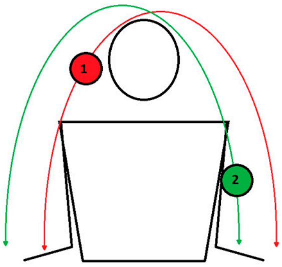<br>**25. The two-handed parabolas with a different focus of attention - catching the second ball**<br>*(see video, Supplemental Digital Content 26, which demonstrates exercise named: "the two-handed parabolas with a different focus of attention – catching the second ball")*	**25.**<br>Two balls, each in different hand. The exercise is very similar to the previous one. Here practitioners should focus on the second hand. It's important to throw the first ball in a perfect parabola trajectory, but the first ball can fall on the ground. The second ball should be thrown in a perfect parabola trajectory, but this time this ball should be caught. Order of exercises: (1) throw the first ball, (2) throw the second ball, (3) the first ball falls on the ground, (4) catch the second ball.

	**26. The two-handed parabolas**<br>*(see video, Supplemental Digital Content 27, which demonstrates exercise named: "the two-handed parabolas")*<br><br>**27. The one-handed column and the parabola**<br>*(see video, Supplemental Digital Content 28, which demonstrates exercise named: "the one-handed column and the parabola")*<br>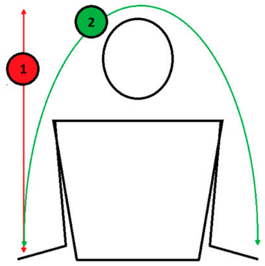	**26.**<br>Two balls, each in different hand. In this exercise practitioners should perform the same movement sequence like in *no.24.* and *no.25.* exercises. But this time it is important to catch both balls.<br><br>**27.**<br>It's a combination of two exercises: column, and parabola. Both balls should be in one hand. Order of the movement: (1) toss the first ball in column, (2) throw the second ball in parabola, (3) catch the first ball, (4) catch the second ball. Column throw should be straight. Both ball should be thrown at the eye-line.
**Fourth unit**	**Practicing:**<br>**14. The two-handed simultaneous columns at three levels**<br>**15. The two-handed simultaneous columns at different levels**<br>**16. The two-handed alternative columns with arms' extensions**<br>**21. The one-handed parabola, with the second ball in the catching hand**<br>**24. The two-handed parabolas with a different focus of attention – catching the first ball**<br>**25. The two-handed parabolas with a different focus of attention - catching the second ball**<br>**26. The two-handed parabolas**<br><br>**Learning:**<br><br>**28. The two-handed alternative columns, catching with one hand**	**Practicing phase**<br>It is a phase of main part of training units, where practitioners perform previously learned exercises.<br><br>**28.**<br>Two balls, each in different hand. This exercise requires to perform alternative

	(see video, Supplemental Digital Content 29, which demonstrates exercise named: "the two-handed alternate columns, catching with one hand")	tossing of two balls. Catching should be performed just by one hand (hand which tosses firstly, should catch both balls). It is important to toss in straight column with both hands.
	**29. The two-ball in one hand column with one ball** *(see video, Supplemental Digital Content 30, which demonstrates exercise named: "the two-ball in one hand column with one ball")*	**29.** Practitioners should keep two balls in one hand. The first ball should be tossed in a straight column trajectory. The second ball should be held constantly in this hand, to make the catching more difficult.
	**30. The two-ball in one hand fountain – one circle** *(see video, Supplemental Digital Content 31, which demonstrates exercise named: "the two-ball in one hand fountain – one circle")* 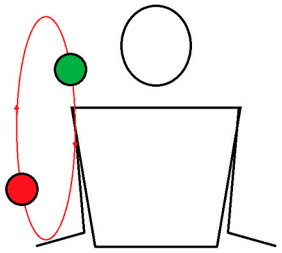	**30.** Two balls in one hand. It is a specific exercise, which is a part of a four-ball juggling exercise. But in this protocol, the main objective of this task is to teach how to throw balls at the right pace. This exercise requires to throw the second ball at the exact moment when the first ball is in the peak of its trajectory. Practitioners should perform this exercise with focus on the throwing. It is not necessary to catch both balls, the second one can fall on the ground.
	**31. The two-ball in one hand fountain – more than one circle** *(see video, Supplemental Digital Content 32, which demonstrates exercise named: "the two-ball in one hand fountain – more than one circle")*	**31.** Two balls in one hand. This is a variation of the previous exercise. Practitioners should throw the balls as many times as it is possible without dropping.
	**27. The one-handed column and the parabola**	**27.** Exercise *no. 27.*
	**32. The one-handed column and the parabola at pace** *(see video, Supplemental Digital Content 33, which demonstrates exercise named: "the one-handed column and the parabola at pace")*	**32.** Two balls in one hand. Modification of *no.27,* which requires from practitioners more dynamic throwing at pace.
	**33. The one-handed column and the parabola with three balls at pace**	**33.** Two balls in one hand, the third ball in the second hand. The same exercise like the previous one, but with another ball in

	(see video, Supplemental Digital Content 34, which demonstrates exercise named: "the one-handed column and the parabola with three balls at pace")<br>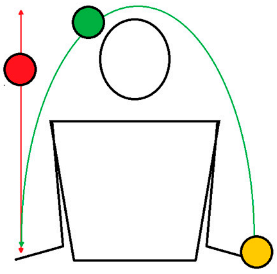	hands. Each ball is used in this exercise. The third ball makes this exercise smoother. Practitioners should start this exercise with the hand with two balls.
**Fifth unit**	**Practicing:**<br><br>**34. The two-handed simultaneous columns**<br>*(see video, Supplemental Digital Content 35, which demonstrates exercise named: "the two-handed simultaneous columns")*<br><br>**35. The two-handed simultaneous columns simultaneously with clapping hands**<br>*(see video, Supplemental Digital Content 36, which demonstrates exercise named: "the two-handed simultaneous columns with clapping hands")*<br><br>**36. The two-handed simultaneous columns with a partner**	**Practicing phase**<br>It is a phase of main part of training units, where practitioners perform various forms of previously learned exercises.<br><br>**34.**<br>Two balls, each in different hand. Similar exercise to *no.14* but each toss should be performed at the medium level (eye-line).<br><br>**35.**<br>Two balls, each in different hand. Variation of the previous task, which requires clapping hands, when a ball is in the air.<br><br>**36.**<br>Two balls, each in different hand. In this group of tasks practitioners practice in pairs. They should be facing each other. When one person in a pair performs exercises, the other, for example, shows numbers by his/her fingers. The task of the person tossing the balls is to say aloud the numbers seen without stopping the exercise. Other version of this task requires tossing balls by both people in a pair. Taking turns, people show funny faces to each other. These faces should be mimicked by the other person without stopping the exercise.

	**37. Fake columns**<br>*(see video, Supplemental Digital Content 37, which demonstrates exercise named: “fake columns”)*<br>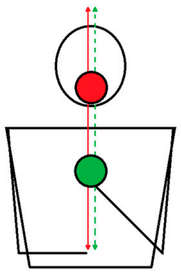	**37.**<br>Two balls, each in different hand. This exercise requires to simultaneously toss one ball and guide the second one with hand along the trajectory of the column. The first version of this task requires guiding the second ball next to the first ball (simulation of the two-handed simultaneous column), the second version depends on guiding the second ball above the first one, and another version requires that guided ball is below the first ball.
	**38. The two-handed alternative columns**<br>*(see video, Supplemental Digital Content 38, which demonstrates exercise named: “the two-handed alternate columns”)*<br><br>**26. The two-handed parabolas**<br>**32. The one-handed column and the parabola at pace**<br><br>**Learning:**	**38.**<br>Two balls, each in different hand. Variation of *no.16.* without extension of the arms.
	**39. The one-handed column and the parabola at pace with tapping on the chest**<br>*(see video, Supplemental Digital Content 39, which demonstrates exercise named: “the one-handed column and the parabola at pace with tapping on the chest”)*	**39.**<br>Two balls in one hand. This exercise should be done in the following order: (1) toss the first ball in a column trajectory; (2) throw the second ball in a parabola trajectory (when the first ball is at the peak of its trajectory); (3) tap with the second hand in the chest. One repetition of this exercise should be ended with one ball in each hand.
	**40. The one-handed column and the parabola at pace with tapping on the chest with three balls**<br>*(see video, Supplemental Digital Content 40, which demonstrates exercise named: “the one-handed column and the parabola at pace with tapping on the chest with three balls”)*	**40.**<br>Two balls in one hand, the third ball in the second hand. This task is similar to the previous one, but here practitioners should hold the third ball in the second hand during exercise. The third ball makes this exercise smoother. Practitioners should start this exercise with the hand with two balls.

	**41. The one-handed column and the parabola at pace with marking**<br>*(see video, Supplemental Digital Content 41, which demonstrates exercise named: "the one-handed column and the parabola at pace with marking")*<br><br>**31. The two-ball in one hand fountain – more than one circle**	**41.**<br>Two balls in one hand, the third ball in second hand. It is a variation of the previous task, which requires marking with the second ball instead of the tap on the chest. Practitioners should start exercise with the hand with two balls.<br><br>**31.**<br>Exercise *no. 31.*
**Six unit**	**Practicing:**<br><br>**26. The two-handed parabolas**<br>**31. The two-ball in one hand fountain – more than one circle**<br>**32. The one-handed column and the parabola at pace**<br>**39. The one-handed column and the parabola at pace with the sequence of three points**<br>**40. The one-handed column and the parabola at pace with the sequence of three points with three balls**<br>**41. The one-handed column and the parabola at pace with the sequence of three points with three balls - marking**<br><br>**Learning:**<br><br>**42. The two-handed parabolas with three balls – throwing two balls**<br>*(see video, Supplemental Digital Content 42, which demonstrates exercise named: "the two-handed parabolas with three balls")*	**Practicing phase**<br>It is a phase of the main part of training units, where practitioners perform previously learned exercises.<br><br>**42.**<br>Two balls in one hand, the third ball in the second hand. Practitioners should perform exercise *no. 26.* with additional – third ball in one hand. It is important to start this exercise with the hand, which holds two balls.

	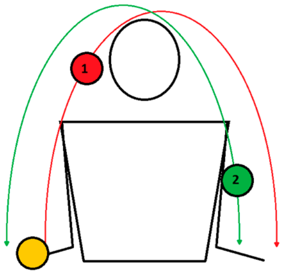	
	**43. The two-handed parabolas with three balls – a tap on the chest**<br>*(see video, Supplemental Digital Content 43, which demonstrates exercise named: "the two-handed parabolas with three balls – a tap on the chest")*	**43.**<br>Two balls in one hand, the third ball in the second hand. It is a variation of the previous exercise, which requires a tap on the chest before the second ball falls into the hand. The order of this task is as follows: (1) throw the first ball; (2) throw the second ball; (3) catch the first ball; (4) tap on the chest; (5) catch the second ball. It is important to start the exercise with the hand, which holds two balls.
	**44. The two-handed parabolas with three balls – marking**<br>*(see video, Supplemental Digital Content 44, which demonstrates exercise named: "the two-handed parabolas with three balls – marking")*	**44.**<br>Two balls in one hand, the third ball in the second hand. It is a variation of the previous task, which requires marking with the second ball instead of the tap on the chest. It is important to start this exercise with the hand, which holds two balls.
	**45. The three-ball parabolas at pace**<br>*(see videos, Supplemental Digital Content 45, Supplemental Digital Content 46 and Supplemental Digital Content 47, which demonstrate the same exercise named: "the three-ball parabolas at pace" but at different levels of difficulty)*	**45.**<br>Two balls in one hand, the third ball in the second hand. In this task practitioners for the first time try to make a full cascade with three balls. Each ball should be thrown in a parabola trajectory at pace, and caught. At this stage the practitioner is not required to perform the three ball cascade. Throwing in a perfect trajectory is more important. This exercise is different from the previous one only in that the hand, which marks, now throws the ball. It is important to start the exercise with the hand, which holds two balls.
	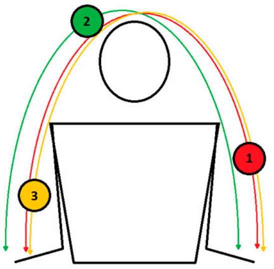	

**Seventh unit**	**Practicing:**<br><br>**30. The two-ball in one hand fountain – one circle**<br><br>**46. The two-handed parabolas with three balls with a different focus of attention**<br>*(see video, Supplemental Digital Content 48, which demonstrates exercise named: "the two-handed parabolas with three balls with a different focus of attention")*<br><br>**47. The two-handed parabolas with three balls with a different focus of attention with marking**<br>*(see video, Supplemental Digital Content 49, which demonstrates exercise named: "the two-handed parabolas with three balls with a different focus of attention with marking")*<br><br>**Learning:**<br><br>**48. The two-ball parabolas, with three balls in hands with march movement**<br><br>**49. The two-ball parabolas, with three balls in hands with the rotation of the body**<br><br>**45. The three-ball parabolas at pace**	**Practicing phase**<br>It is a phase of the main part of training units, where practitioners perform various forms of previously learned exercises.<br><br>**46.**<br>Two balls in one hand, the third ball in the second hand. This exercise is similar to *no.24.* and *no.25.* In this variation practitioners should focus on the one ball (the first, second, or third). Each ball should be thrown in a perfect parabola trajectory at the eye-level, but two balls can fall on the ground. Practitioners should catch only the ball, which they focused on. It is important to start the exercise with the hand, which holds two balls.<br><br>**47.**<br>Two balls in one hand, the third ball in the second hand. This task is similar to the previous one. In this task practitioners should make a marking before they catch the ball, which they focused on. This means that only two balls are thrown. It is important to start the exercise with the hand, which holds two balls.<br><br>**48.**<br>Two balls in one hand, the third ball in the second hand. In this task practitioner should perform like *no.42.* task, with marching three steps back and forth. This exercise is aimed at correcting the trajectory of the balls. It is important to start the exercise with the hand, which holds two balls.<br><br>**49.**<br>Two balls in one hand, the third ball in the second hand. It is a variation of the previous task, but in this case, practitioners should rotate around their axis while performing *no.42.* It is important to start the exercise with the hand which holds two balls.<br><br>**45.**<br>Exercise *no.45.*

Eight unit	Practicing & learning:	Practicing & learning phase
	**Practicing & learning:**	**Practicing & learning phase**<br>Practitioners perform variations of previously learned exercises, to develop the ability to juggle with three balls.
	**42. The three balls – two hands parabolas**	**42.**<br>Exercise *no.42*.
	**50. The two-handed parabolas with a different focus of attention - catching the last ball**<br>*(see video, Supplemental Digital Content 50, which demonstrates exercise named: "the two-handed parabolas with a different focus of attention – catching the last ball")*	**50.**<br>Two balls in one hand, the third ball in the second hand. It is a variation of one of the exercises with different focus of attention – *no.46*. Practitioners should follow this order: (1) throw the first ball; (2) throw the second ball; (3) ignore the first ball; (4) throw the third ball; (5) ignore the second ball; (6) catch the third ball. Each ball should be thrown in a perfect parabola trajectory and at the eye level. It is important to focus only on the last ball. It is important to start the exercise with the hand, which holds two balls.
	**51. The two-handed parabolas with a different focus of attention - catching the last two balls**<br>*(see video, Supplemental Digital Content 51, which demonstrates exercise named: "the two-handed parabolas with a different focus of attention – catching the last two balls")*	**51.**<br>Two balls in one hand, the third ball in the second hand. It is a variation of the previous task. The difference is that, practitioners should additionally catch the second ball at the point (5), as described previously in exercise n*o.50*. It is important to start the exercise with the hand, which holds two balls.
	**52. The two-handed parabolas with a different focus of attention - catching the first ball**<br>*(see video, Supplemental Digital Content 52, which demonstrates exercise named: "the two-handed parabolas with a different focus of attention – catching the first ball")*	**52.**<br>Two balls in one hand, the third ball in the second hand. In this variation of exercise with different focus of attention, practitioners should catch only the first ball. The order is as follows: (1) throw the first ball; (2) throw the second ball; (3) catch the first ball; (4) throw the third ball; (5) ignore the second ball; (6) ignore the third ball. A perfect trajectory still should be required. It is important to start the exercise with the hand, which holds two balls.
	**53. The two-handed parabolas with a different focus of attention - catching the first two balls**	**53.**<br>Two balls in one hand, the third ball in the second hand. This task is similar to the previous one. , practitioners should

	(see video, Supplemental Digital Content 53, which demonstrates exercise named: "the two-handed parabolas with a different focus of attention – catching the first two balls)<br><br>**54. The cascade flash**<br><br>**46. The three-ball parabolas at pace**<br><br>**55. The cascade flash with marching**	additionally catch the second ball at the point (5), as described previously in exercise *no.52*. It is important to start the exercise with the hand, which holds two balls.<br><br>**54.**<br>This exercise is similar to *no.45.* But this task requires one throw and one catch of each ball. Practitioners should throw balls at the eye level in a perfect parabola trajectory.<br><br>**46.**<br>Exercise *no.46.* At this stage it is an attempt to perform a three ball cascade as long as possible. In other words, it is the previous exercise, but performed in a sequence.<br><br>**55.**<br>Variation of exercise *no.54.* Practitioners should perform this juggling exercise marching three steps back and forth.
**Ninth unit (just practicing of three-ball cascade)**	**Practice:**<br>**46. The three-ball parabolas at pace**	**Practicing phase**<br>Exercise *no.46.* The ninth unit is only about practicing the three-ball at pace cascade. Practitioners should try to achieve a flash of three balls. They can be guided by the instructor to perform some of previous exercises, which should help them achieve the main objective.
**Tenth unit**	**Practice & learning:**<br>**56. The one-handed and one ball column and the parabola with three balls**<br>*(see video, Supplemental Digital Content 54, which demonstrates exercise named: "the one-handed and one ball column and the parabola with three balls")*<br>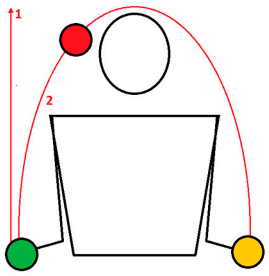	**56.**<br>Two balls in one hand, the third ball in the second hand. Two balls should be held during the whole exercise. Only one ball is thrown. Task should be performed in the following order: (1) toss the ball in a column trajectory; (2) catch the ball; (3) throw the ball in a parabola trajectory; (4) catch the ball. It is important to start the exercise with the hand, which holds two balls.

	**40. The one-handed column and parabola in pace with three points of sequence with three balls**	**40.**<br>Exercise *no.40.*
	**57. The two-ball in one hand columns**<br>*(see video, Supplemental Digital Content 55, which demonstrates exercise named: "the two-ball in one hand columns")*<br>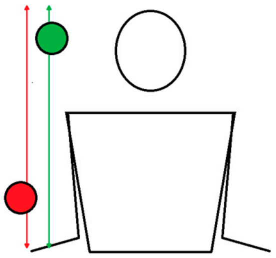	**57.**<br>Two balls in one hand. This exercise is very similar to *no.30.* But here practitioners shouldn't throw balls in circle. Tossing should be performed in a perfect and straight column trajectory.
	**58. The two-ball in one hand columns with catching in both hands**<br>*(see video, Supplemental Digital Content 56, which demonstrates exercise named: "the two-ball in one hand columns with catching in both hands")*	**58.**<br>Two balls in one hand. Easier variation of the previous exercise. It is important to throw balls in a perfect straight trajectory. The second ball should be caught by the second hand (the hand must reach for the ball). An additional complication can be to hold the second hand behind the back, before grabbing the ball or hold the third ball in the second hand.
	**59. The two-ball in one hand columns, three balls, catching two balls in both hands, releasing the third ball**<br>*(see video, Supplemental Digital Content 57, which demonstrates exercise named: "the two-ball columns, three balls, catching two balls in both hands, releasing the third ball")*	**59.**<br>Two balls in one hand, the third ball in the second hand. This exercise requires releasing one of three balls. Order of this exercise should be as follows: (1) toss the first ball in a column trajectory; (2) toss the second ball (with the same hand as in point 1) in a column trajectory; (3) release the third ball on the ground; (4) catch the first ball in the same hand; (5) catch the second ball in the hand that let go of the ball. In these exercise, the second toss, can be in a parabola trajectory. It is important to start the exercise with the hand, which holds two balls.
	**60. The two-ball in one hand columns, three balls,**	**60.**<br>Two balls in one hand, the third ball in the second hand. Another variation of the previous task. In this exercise practitioners

	**catching two balls in both hands, tossing the third ball** *(see video, Supplemental Digital Content 58, which demonstrates exercise named: "the two-ball in one hand columns, three balls, catching two balls in both hands, tossing the third ball")*<br><br>**61. The three balls "W"** *(see video, Supplemental Digital Content 59, which demonstrates exercise named: "the three balls W")*<br><br>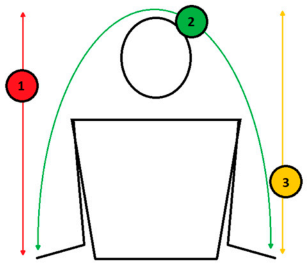<br><br><br>**Practice:**<br><br>**45. The three-ball parabolas at pace**	should toss the third ball in a column trajectory, before they catch the second ball. The third ball can fall on the ground. It is important to start the exercise with the hand, which holds two balls.<br><br>**61.**<br>Two balls in one hand, the third ball in the second hand. This task requires a smooth movement of each ball. It is not necessary to master this exercise in a smooth form (when none of the balls come into contact with each other). Order of this exercise for smooth version should be as follows: (1) toss the first ball in a column trajectory; (2) throw the second ball in a parabola trajectory; (3) catch the first ball; (4) throw the third ball in a column trajectory; (5) catch the second ball; (6) another throw of the second ball in a parabola trajectory; (7) catch the third ball. It is important to start the exercise with the hand, which holds two balls.<br><br>**Practicing phase:**<br><br>**45.**<br>Exercise *no.45.* Practitioners should try to achieve a flash of three balls. They can be guided by the instructor to perform some of previous exercises, which should help them achieve the main objective.
**Eleventh unit**	**Practice & learning:**<br><br>**62. The zero throw** *(see videos, Supplemental Digital Content 60 and the Supplemental Digital Content 61, which demonstrate exercise named: "the zero throw" but at different levels of difficulty)*	**62.**<br>It is a simple exercise, which requires throwing one ball between hands in a horizontal trajectory. This throw should be very dynamic. At this stage this exercise should be performed by throwing the ball from hand to hand twice very quickly. Practitioners should avoid a direct transfer of the ball.

	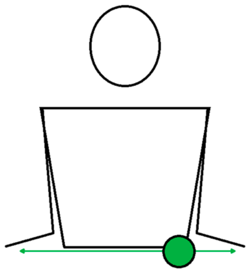	
	**63. The one-ball box** *(see video, Supplemental Digital Content 62, which demonstrate exercise named: “the one-ball box”)*	**63.** It is a simple combination of a zero throw and a column with one ball. Practitioners should follow this order: (1) throw the ball in a column trajectory and catch it; (2) throw the ball in a horizontal trajectory and catch it in another hand; (3) throw the ball in a column trajectory and catch it.
	**64. The zero throw – two balls** *(see video, Supplemental Digital Content 63, which demonstrate exercise named: “the zero throw – two balls”)*	**64.** Two balls, each in different hand. One ball should be thrown in a horizontal trajectory. The second ball should be held in the hand constantly, to make a catch more difficult.
	**38. The two-handed alternative columns**	**38.** Exercise *no.38.*
	**65. The zero throw and the column at pace – catching two balls in one hand** *(see video, Supplemental Digital Content 64, which demonstrates exercise named: “the zero throw and the column at pace – catching two balls in one hand”)*	**65.** Two balls, each in different hand. This task requires to follow the order: (1) throw the first ball in a column trajectory; (2) throw the second ball in a horizontal trajectory and catch it in another hand; (3) catch the first ball. One repetition of this task should be ended with two balls in one hand.
	**66. The zero throw and the column at pace – catching two balls in two hands** *(see video, Supplemental Digital Content 65, which demonstrates exercise named: “the zero throw and the column at pace – catching two balls in two hands”)*	**66.** This task is similar to the previous one. But here practitioners should end the task with two balls in two hands. The zero throw should be done twice and very dynamically. Practitioners should follow the order: (1) throw the first ball in a column trajectory; (2) throw the second ball in a horizontal trajectory twice; (3) catch the first ball.
	**67. The two-ball box** *(see video, Supplemental Digital Content 66, which demonstrates exercise named: “the two-ball box”)*	**67.** It is an exercise, which requires the same throwing task as in the previous exercise. But one throw is performed by one side of the body, one by the other one. To make

	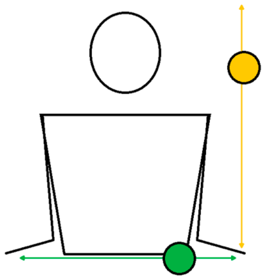 **Practice:** **45. The three-ball parabolas at pace**	the task more difficult practitioner may try to make throws and catches at the same time with both hands. **Practicing phase** **45.** Exercise *no.45.* Practitioners should try to achieve a flash of three balls. They can be guided by the instructor to perform some of previous exercises, which should help them achieve the main objective.
**Twelfth unit (just practicing of three ball cascade)**	**Practice:** **45. The three-ball parabolas at pace**	**Practicing phase** **45.** Exercise *no.46.* The twelfth unit is only about practicing the three-ball at pace cascade. Practitioners should try to achieve a flash of three balls. They can be guided by the instructor to perform some of previous exercises, which should help them achieve the main objective. In this unit it is important to achieve the highest possible number of tosses and catches.

Calm down

It was a regular part of the training units. The purpose of the calm down was to lower arousal, as well as to stretch. Examples of exercises used in calm down part of the unit are listed below:

- Breath control
- Lifting the toes with shoulder abduction
- Arms out to the side and back to stretch the chest
- Arm circles
- Side bends
- Torso rotation
- Forward bend
- Cat stretch flexion in standing position
- Neck stretch
- Shoulder stretch
- Etc.
